# The Institutional Library

**Published:** 1916-02-05

**Authors:** 


### THE INSTITUTIONAL LIBRARY.

### The Rise of Rail-Power in War and Conquest—1833 to 1914.

By Edwin A. Pratt.

(London: P. S. King and Sons, Ltd., Orchard House, Westminster. Price 7s. 6d. net.)

The title of this book does not give much promise of being of interest to the general reader, but as a matter of fact it is one of those books which, opened anywhere, will always be found interesting.

In Chapter VIII., "Railway Ambulance Transport," we learn that the earliest occasion on which the railway was made use of for the conveyance of sick and wounded from a scene of actual hostilities to the rear was the Crimean War, when the military line between Balaklava and the camp before Sebastopol was so employed. The facilities afforded were, however, of the most primitive character, and the sufferings of the wounded on the journey must often have been very great. The development of the present hospital train is traced through the various wars, since 1860, in America, Europe and South Africa.

The author goes very fully into the subject of his book—namely, the evolution and development of rail power in war and conquest, and quotes almost every book that appears to have been written upon or connected with railways in war. We read there were many military sceptics in the early days who, basing their calculations on locomotive performances of their time, asserted that, although the railway might be of service in the conveyance of supplies, guns, and ammunition, it would be of no advantage in the transport of troops. In 1847 one of the leading military writers in Germany published a pamphlet in which he sought to prove that the best organised railway could not carry 10,000 infantry a distance of sixty miles in twenty-four hours. As regards the conveyance of cavalry and artillery by train, he declared that this would be an impossibility. In Chapters XIX. and XX., "A German African Empire" and "Designs on Asiatic Turkey," Mr. Pratt suggests that the German strategical railways in their African colonies were avowedly constructed to lead the way to the creation of a German African Empire, and that the Baghdad Railway was designed to ensure the establishment of a German Middle-Asian Empire, bringing under German control the entire region from the Mediterranean to the Persian Gulf, and providing convenient stepping-off places from which an advance might be made on Egypt in the one direction and India in the other. He concludes from the various facts quoted that one, at least, of the main objectives of Germany in provoking the Great World War was no less a prize than the African continent, and that when she invaded Belgium and France she did so less with the object of annexing the former country and of creating another Alsace-Lorraine in the latter than of having "something in her hand" with which to "bargain" in the interests of her projects in Africa when the time came for discussing the terms of peace, assuming that she had not already attained her purpose at the outset by sheer force of what she thought would be her irresistible strength.

---

**English Public Health Administration.** By B. G. Bannington. With an Introduction by Graham Wallas. (London: P. S. King and Sons, Limited. Price 7s. 6d. net.)

The ai[illegible] of this book is to fill a place between that occupied b[illegible] the practical handbook for the sanitary officer and that of the digest of Acts of Parliament. The volume, therefore, deals with the administrative organisation of the Public Health Department, which is separately considered as a unit of our Local Government system. While each chapter is complete in itself, and discusses such subjects as the medical officer of health, the right of entry, hospitals and their administration, and the control of tuberculosis, the effect of the whole is to give the reader a luminous and many-sided picture of the various machinery by which our "public health system" is organised. The whole of this branch of work is so new, and the beginnings of co-ordination are so recent, that it is perhaps surprising that there is not more overlapping and obstruction than is actually the case. Every branch of the work is growing, and all intelligent and public-spirited people must hope that our public health administration is sufficiently established to make a set-back to its development impossible. Institutional workers will turn with lively interest to Mr. Bannington's pages, and gain from them a useful, lucid, and indeed inspiring account of the achievements and working of English public health administration. Hospital readers in particular will not fail to note the broad-mindedness with which a public official points to the value of voluntary organisations, and to the useful part they play, not merely by their institutions, but as pioneers and instigators of fresh activity in the local authorities with whom they co-operate. The last chapter, entitled "The Need for Reform," is full of interest, and in it Mr. Bannington contrasts the completeness of organisation which exists for the discovery and prevention of nuisances with the confusion obtaining among the authorities for the treatment of disease. In preventive organisation since the Royal Sanitary Commission of 1870 much improvement has been made, but he urges that "the establishment of a public medical service . . . centering upon and directed from the public health department of the local authority, is urgently called for." He does not advocate a central Department of Public Health, with a Cabinet Minister at its head, on the ground that the multiplication of central departments, with powers of local interference, is a mistake to avoid. We have said enough to show that "English Public Health Administration" is an able and informing piece of work, which will give to many workers in this varied field of activity a better idea than they have hitherto had of their relation to each other, and of the desirability of co-ordinating into a true system the complexities and scattered efforts of our present organisations for the treatment as well as the prevention of disease.

**Medical Emergencies.** By JAMES RAE, M.A., M.D. The S.P. Pocket Guide Series. (London: The Scientific Press. Price 1s. net.)

As long as there are available such compact and excellent little text-books as those of the S.P. Pocket Guide Series nurses cannot complain of any lack of facilities for learning the why and wherefore of various forms of treatment. In this, the latest addition to the series, it is explained how emergencies may arise in the course of disease and how they may be met in accordance with the most approved methods of modern practice. To cover the whole range of medical emergencies in so small a compass it has been necessary for Dr. Rae to write dogmatically and concisely; but it will be found that the procedures advised are described at sufficient length to enable an intelligent nurse to follow their *rationale*, and to carry out such as come within her sphere, or to prepare for those forms of treatment in which she will be only called upon to assist. Many of the details in this book will be new to a considerable number of readers, for the author has avoided the least tendency to describe old and hackneyed methods where more up-to-date ones have proved efficacious in his opinion. This little manual is sure to be one of the most popular of the series.

**Instinct and Intelligence.** By N. C. MACNAMARA, F.R.C.S. (London: Henry Frowde and Hodder and Stoughton. Price 6s. net.)

IT is curious at this time of day to read a book on the subject of instinct and intelligence without meeting in it any recognition of the profound insight and originality which Samuel Butler brought to bear on their elucidation. Hering, doubtless because he was a foreigner and not an Englishman, receives bare mention, but there is nothing to show that Mr. Macnamara is acquainted with Butler's famous definition of instinct as "inherited memory," compared to which the definition which *is* quoted is as dust and ashes in the mouth. However, though Butler's extraordinary clarity of thought is not made use of in these pages, the writer has this excuse, if excuse it be, that his aim is rather to remind the educationists that they have to train not only the mind, but the instincts which result from the nervous substance of the brain itself, than to explain the nature of instinct. He therefore gives a detailed account of the development of the nervous system from the simplest organisms to man. The way in which scattered ganglia are gradually merged so as to form the foundations of the brain, with its eventual covering, the "neopallium," is described at length; but when it comes to applying this knowledge to the practical work of education the author is not fruitful in suggestion. Indeed he is in the main content to pass by this aspect of his subject, merely recommending the books of Edith Reed Mumford and M. A. Wood. The book, then, is readable for the physiological and biological material that it contains rather than as a guide to the principles which should underlie education. But while admitting the interest and value of acquainting teachers with the nervous mechanism that lies at the root of instinct and intelligence, any reader who wishes to understand the relation of these to each other, their limitations and the respective parts that they play in evolution, would do better to read "Life and Habit" and "Unconscious Memory."

**The Seven Ages of Woman.** By MARY SCHARLIEB, M.D., M.S. (London: Cassell and Co. 1915. Pp. 286. Price 6s. net.)

THERE are many books devoted to the advice of the wife and mother upon the care of her own health and that of her family; and it is a sign of the times that on the whole they reach a very fair level of excellence. In adding one more to the class Mrs. Scharlieb writes with a personal and authoritative style which would command attention were she an unknown authoress instead of a leading medical woman of the day. A certain sloppiness is very common in this type of book, and Mrs. Scharlieb does not avoid it altogether, though she is well ahead of most other authoresses in this respect. Her advice is sound, if occasionally a little vague; and her large experience of medical practice has obviously helped her in the composition of a thoroughly sound and sympathetic text-book of gynæcosophy.

**The Structure of the Fowl.** By O. CHARNOCK BRADLEY, M.D., D.Sc., M.R.C.V.S. With 73 Illustrations. (London and Edinburgh: A. and C. Black. Price 3s. 6d. net.)

CONSIDERING the relatively small size of this book—and it runs to barely 150 pages—it contains a remarkable amount of detailed information on the subject of the anatomy and physiology of the domestic fowl. The poultry-keeper, whose name is now legion, will be able to gain from it a clear understanding of the structure and normal health conditions of the bird, while at the same time the student, professional or lay, who has to investigate the diseases to which poultry are subject will be stimulated and enlightened by the account of the microscopic structure of its organs, which is set forth with a generous amount of illustration. Forming one of the Edinburgh Medical Series, Dr. Bradley's book is a handbook for the student, but every poultry-keeper who realises the importance of scientific knowledge would be well repaid by mastering its contents, in which fullness of detail and clearness of exposition are combined.

**The Wounded French Soldier.** By DION CLAYTON CALTHROP. (The St. Catherine Press, 34 Norfolk Street, Strand. Price 1s. 6d. net.)

THIS little book, which is published in aid of the French Red Cross (Comité de Londres), is a series of impressions of France at war, as seen behind the trenches by an Englishman, who is either a combatant himself or a member of a Red Cross unit. He tells us he was wounded, and what his impressions were on watching the ruins of Rheims, the wounded on the road and in hospital, or the maimed making artificial flowers, and so on. There is a conscious literary flavour about these pages—an artifice, indeed, which reminds us that Mr. Calthrop is an author, the historian, if our memory serves us, of the harlequinade, and the joint-author with Granville Barker of the historical section of the harlequinade which preceded that wonderful play, "Androcles and the Lion," two years ago at the St. James's Theatre. His impressions, then, have an air about them and catch attention, but they make us ask ourselves why it is that writers on the war, who have seen its activities at first hand, seem to have to strain and stretch to give an impression of its awfulness? The book is very nicely printed, and contains many illustrations, some of which show what shells can do in the way of destruction. Anyone who begins to read it will certainly finish it; and the cause for which it is written leads us to hope that its sale not only will be large, but that the subscriptions it will attract will be even larger. The London address of the French Red Cross is 9 Knightsbridge, S.W.

